# Dr. Richard Beverly Cole

**Published:** 1901-02

**Authors:** 


Dr. Richard Beverly Cole, of San Francisco, Cal., died in that city January 17, 1901, aged 71 years. Dr. Cole was a graduate of Jefferson Medical College, class of 1848, and had been practising and teaching his profession for over 50 years. He was a genial companion and an accomplished physician.

